# Plant hormones regulate inducible asexual reproduction in *Kalanchoë pinnata*

**DOI:** 10.1093/jxb/eraf405

**Published:** 2025-09-16

**Authors:** Francisco Jácome-Blásquez, Joo Phin Ooi, Itzel M Viveros-Sánchez, Victoria Spencer, Yiğit Berkay Gündoğmuş, Minsung Kim

**Affiliations:** Division of Molecular and Cellular Function, School of Biological Sciences, Faculty of Biology, Medicine and Health, The University of Manchester, Manchester, M13 9PT, UK; Division of Molecular and Cellular Function, School of Biological Sciences, Faculty of Biology, Medicine and Health, The University of Manchester, Manchester, M13 9PT, UK; Division of Molecular and Cellular Function, School of Biological Sciences, Faculty of Biology, Medicine and Health, The University of Manchester, Manchester, M13 9PT, UK; Division of Molecular and Cellular Function, School of Biological Sciences, Faculty of Biology, Medicine and Health, The University of Manchester, Manchester, M13 9PT, UK; Division of Molecular and Cellular Function, School of Biological Sciences, Faculty of Biology, Medicine and Health, The University of Manchester, Manchester, M13 9PT, UK; Division of Molecular and Cellular Function, School of Biological Sciences, Faculty of Biology, Medicine and Health, The University of Manchester, Manchester, M13 9PT, UK; University College Dublin, Ireland

**Keywords:** Asexual reproduction, *Kalanchoë*, leaf crenulations, plant hormones, plantlet formation, somatic embryogenesis

## Abstract

Many *Kalanchoë* species reproduce asexually by forming plantlets in the leaf margins. Plant hormones are involved in meristem and embryogenesis pathways, yet their exact role in *Kalanchoë* plantlet formation is unknown. Here, we show that auxin and cytokinin-mediated pathways facilitate the establishment of leaf crenulations and, in turn, plantlet primordia in *K. pinnata*. Antisense transgenic lines with lower expression of the auxin transporter *PIN-FORMED1* (*PIN1*) and the cytokinin signalling inhibitor *ARABIDOPSIS HISTIDINE-CONTAINING PHOSPHOTRANSFER PROTEIN* (*AHP*) generated fewer plantlets. *GREEN FLUORESCENT PROTEIN* (*GFP*) reporter lines visualising the activity of auxin and cytokinin also revealed the accumulation of these hormones in the plantlet primordia. Furthermore, treatment of leaves with gibberellic acid (GA_3_) prevented plantlet emergence, suggesting that its depletion activates plantlet formation in detached leaves. Our findings highlight the involvement of hormonal regulation in the inducible plantlet formation system, which facilitates the formation of plantlet primordia and exerts precise control over dormancy processes in detached leaves. This study provides insight into the role of hormones in the novel reproductive system in the genus *Kalanchoë*.

## Teaser

Auxin, cytokinin, and GA_3_ interact to regulate the formation and development of plantlets on the leaves of *Kalanchoë pinnata*, with GA_3_ maintaining dormancy of epiphyllous buds on attached leaves.

## Introduction

Many *Kalanchoë* species regenerate offspring along their leaf margins (Allorge-Boiteau, 1996; Garces *et al*., 2007). Derived *Kalanchoë* species, such as *K. daigremontiana*, have relinquished their capacity for sexual reproduction, instead relying mostly on the formation of plantlets for reproduction (Garces *et al*., 2007). Another subset of *Kalanchoë* exclusively generates plantlets under stress conditions, such as leaf detachment from the mother plant. Intriguingly, these species retain the capability for sexual reproduction (Allorge-Boiteau, 1996; Garces *et al*., 2007). Molecular studies have confirmed that plantlet formation in *Kalanchoë* has co-opted organogenesis and embryogenesis pathways (Garces *et al*., 2007; Jácome-Blásquez *et al*., 2022; Jácome-Blásquez and Kim, 2023). *Kalanchoë SHOOT MERISTEMLESS* (*STM*) orthologs have been found to be ectopically expressed in the leaves of various plantlet-forming species in the genus (Garces *et al*., 2007; Jácome-Blásquez and Kim, 2023). *STM* is known to control shoot apical meristem (SAM) development and maintenance in Arabidopsis (Long *et al*., 1996). In *K. pinnata*, *STM* and *CUP-SHAPED COTYLEDON 2* (*CUC2*) interact to facilitate the formation of leaf crenulations and promote the ectopic expression of other meristem genes, namely *WUSCHEL* (*WUS*) and *CLAVATA* (*CLV*) (Jácome-Blásquez and Kim, 2023). Moreover, down-regulation of the *STM* ortholog in *K. daigremontiana* (Garces *et al*., 2007) and *K. pinnata* (Jácome-Blásquez and Kim, 2023) severely prevents plantlet formation, suggesting its role in establishing and maintaining pluripotent cells in leaf notches where plantlet primordia develop.

Plant hormones regulate various life-cycle stages, including the formation of the SAM (Davies, 2010). Polar transport of auxin regulates growth at the embryonic stages, orchestrates post-embryonic organ formation, and controls tropism (Blilou *et al*., 2005; Bohn-Courseau, 2010). At the 16–32-cell stage of embryogenesis, PINFORMED7 (PIN7) reverses the auxin flow from apical to basal regions, triggering root apical meristem (RAM) development (Zhou and Luo, 2018). The transport of auxin to the suspensor cell by PIN1 and PIN4 efflux transporters further promotes RAM development (Bohn-Courseau, 2010; Zhou and Luo, 2018). Conversely, lower levels of auxin are required for the development and maintenance of the SAM (Zhang *et al*., 2017; Shi *et al*., 2018); It has been shown that high auxin levels result in the formation of an enlarged SAM (Zhang *et al*., 2017). Any fluctuation in the auxin gradient or a restricted supply due to inhibition or mutations in transporters can result in the formation of excessive axillary buds (Wang *et al*., 2014).

An increase in endogenous auxin levels has been observed at the respective early and induction stages during zygotic and somatic embryogenesis in various plant species (Nic-Can and Loyola-Vargas, 2016). Furthermore, a group of YUCCA (YUC) auxin biosynthesis enzymes involved in somatic embryogenesis is active during zygotic embryogenesis (Radoeva *et al*., 2019). The loss of function in *YUC* genes can result in serious developmental defects, including failure of embryo development (Bai *et al*., 2013). The PIN1 auxin transporter is also essential for maintaining the auxin gradient during somatic and zygotic embryogenesis (Vieten *et al*., 2005). Collectively, this evidence highlights the significance of polar auxin transport for the normal development of zygotic or somatic embryos, and any disturbance can result in serious developmental abnormalities (Abrahamsson *et al*., 2012).

Cytokinin governs cell-to-cell auxin transport by affecting the expression of several *PIN* genes, consequently regulating auxin distribution (Pernisova *et al*., 2016). Cytokinin also modulates local auxin metabolism (Casanova-Sáez *et al*., 2021). Rosspopoff *et al*. (2017) have presented evidence regarding the involvement of cytokinin in the conversion of lateral root primordia to shoots by inducing *WUS* and *CLV3* expression. Furthermore, cytokinin-deficient mutants have failed to exhibit typical SAM formation, thus elucidating the homeostatic role of cytokinin in SAM development (Chickarmane *et al*., 2012). Cytokinin maintains SAM homeostasis by activating type-B *ARABIDOPSIS RESPONSE REGULATORs* (*ARR*s), which promote *WUS* expression by binding to its promoter (Zhao, 2010; Meng *et al*., 2017). ARRs also prevent auxin accumulation and repress *YUC* genes (Meng *et al*., 2017). Cytokinin initiates SAM organ development through the activity of a signalling inhibitor, ARABIDOPSIS HISTIDINE-CONTAINING PHOSPHOTRANSFER PROTEIN 6 (AHP6) (Besnard *et al*., 2014), and maintains a stem cell niche by up-regulating *WUS* expression in the organising centre of the SAM (Zhao, 2010).

Auxin and cytokinin exhibit a concentration-dependent effect on plantlet formation in *Kalanchoë* species (Sawhney *et al*., 1994; Kulka, 2008). *In vitro* studies on *K. daigremontiana* have shown that the application of auxin at low concentrations induce plantlet formation in excised leaves, whereas higher concentrations show inhibitory effects (Yazgan and Vardar, 1977). Cytokinin promotes plantlet formation in *K. daigremontiana* under short-day conditions and in attached leaves of *K. pinnata*, whereas auxin inhibits plantlet formation in both species (Yazgan and Vardar, 1977); however, cytokinin shows inhibitory effects on plantlet formation in *K. marnieriana* (Kulka, 2006). The indole-3-acetic acid (IAA) oxidase cofactor p-coumaric acid induces plantlet formation in *K. pinnata* (Houck and Rieseberg, 1983). This suggests that the signalling mechanism of auxin and cytokinin during plantlet formation is dependent on various interrelated factors and is species-specific.

Gibberellin (GA) promotes embryo growth and radicle protrusion from the seed coat (Olszewski *et al*., 2002), and after germination it enhances stem elongation, leaf expansion, and root growth (Olszewski *et al*., 2002). The level of bioactive GAs and their homeostasis is maintained by tight regulation of *GA2-oxidase* (*GA2ox*) and *GA3-oxidase* (*GA3ox*) (Li *et al*., 2019). *GA2ox2* is usually expressed in the above-ground tissues, and it inactivates biologically active GA_3_ through a hydroxylation mechanism (Li *et al*., 2019). Overexpression of various *GA2ox* genes inhibits hypocotyl elongation in Arabidopsis (Li *et al*., 2019). GA seems to be involved in plantlet formation as external application of GA in *K. delagoensis* (syn. *K. tubiflora*) inhibits plantlet formation (Dostál, 1970). Given that plantlet-forming *Kalanchoë* species express the *STM* ortholog in the plantlet primordia (Garces *et al*., 2007; Jácome-Blásquez and Kim, 2023) and reduced GA_3_ activity promotes meristematic activity through *STM* in Arabidopsis (Hay *et al*., 2002), *GA–STM* pathways might be involved in plantlet formation. However, GA_3_ treatment in *K. daigremontiana* does not affect plantlet formation and only leads to the elongation of the plantlet internodes (Garces *et al*., 2014).

This study tested the hypothesis that auxin, cytokinin, and GA signalling play different roles in the formation of the epiphyllous buds (EBs) from which new plantlets are generated, and in breaking EB dormancy upon leaf detachment in *K. pinnata*. To this end, we manipulated hormone pathways and accumulation using external applications of hormones and hormone inhibitors, as well *via* down-regulation of the expression of *PIN1*, *AHP*, and *GA2ox2* to disrupt the accumulation of auxin, cytokinin, and GAs in the leaf crenulations. We also examined whether effects of external hormone applications were concentration-dependent. As auxin and cytokinin are crucial for embryogenesis and meristematic activities, we would expect the antisense (AS) plants to show fewer plantlets. *Kalanchoë pinnata* represented an ideal species for this study as EBs are formed during leaf development but plantlet initiation occurs only after detachment, thus allowing us to investigate the two events separately. Insights gained in *K. pinnata* can be extended to species in which plantlets are constitutively formed without EB dormancy. Our results showed that the *PIN1* and *AHP* AS plants exhibited round leaves with a reduced number of leaf crenulations and thus fewer plantlets, whilst depletion of GAs might be needed for plantlet initiation. In addition, expression analysis of other hormone genes in the *PIN1*, *AHP*, and *GA2ox2* AS backgrounds suggested possible genetic networks of hormone genes controlling the process of plantlet formation.

## Materials and methods

### Plant materials and growth conditions

The *Kalanchoë pinnata* wild type (WT), reporter lines, and antisense (AS) plants were grown in 1 l pots with a 6:1:1 mix of Levington’s F2 compost (Scott’s Miracle-Gro, UK), perlite (Sinclair Horticulture, Ltd, UK), and vermiculite (Sinclair Horticulture, Ltd, UK). We generated ∼20–30 independent AS lines per gene and selected three lines per gene for further investigation in most experiments. The plants were grown in a Percival Scientific AR-60L growth chamber at 23 °C, illuminated with fluorescent lights under long-day conditions of 16/8 h at 680 lux (12 μmol m^–2^ s^–1^). For the hormone treatment experiments, fully-grown mature leaves (the fourth internode below the shoot) of 3-month-old WT, AS, and GFP reporter plants were used (grown from plantlets: note that these plants are still juvenile with simple leaves). For reporter lines, emerging leaves and developing young leaves (2–3 cm in size) of the shoot apical meristem as well as detached mature leaves (3–21 d after detachment) were used. Several 1 cm^2^ explants were dissected from young, fully-grown whole leaves and used for plant transformation. For RT-qPCR, we used tissues from leaf margins smaller than 0.5 cm for the RNA extraction.

### Image acquisition

Developing plantlets were imaged using a GXCAM-Eclipse (0654) Wi-Fi camera, attached to a Leica S8 APO Stereo Microscope. Images of whole leaves were taken with a 12-megapixel iPhone camera (ultrawide aperture ƒ/2.4; wide ƒ/1.6; telephoto ƒ/2.2). The crenulations of the GFP reporter lines were imaged with a DFC310 FX camera set up with an M165 FC stereo fluorescence microscope (both Leica).

### Hormone treatments

Leaves were cut from WT and AS plants, placed in Ziplock bags, and submerged completely overnight in solutions of 10, 25, 50, and 100 μM of IAA, the synthetic cytokinin thidiazuron (TDZ), gibberellic acid GA_3_, and the GA_3_ antagonist paclobutrazol (PBZ; all Sigma). The controls were 1% methanol, 0.5% Tween-20, and 1% DMSO for IAA, GA_3_, and PBZ, and distilled H_2_O for TDZ. The next day the leaves were removed from the bags and, without rinsing, placed on a dry white paper sheet and kept in a growth chamber under the conditions previously described by Jácome-Blásquez and Kim (2023). For comparison, untreated leaves freshly detached from plants were also placed on the paper sheets. The plantlet emergence was scored every 3 d for 21 d. New plantlets were scored when they became visible, at ∼0.5 mm in length. A total of 25 leaves per treatment were used for this experiment (five independent lines, five leaves per line).

### Gene cloning and vector assembly

Clones of *K. pinnata AHP*, *GA2ox2*, and *PIN1* were isolated using specific primers based on their orthologs from *K. laxiflora* and *K. fedtschenkoi* sequences found in Phytozome v.12.1 (https://phytozome-next.jgi.doe.gov/; Supplementary Table S1). To create the AS constructs, we cloned a 223 bp fragment of *KpAHP* located in two exons from cDNA, a 338 bp fragment of *KpGA2ox2* in exon 1, and a 171 bp fragment of *KpPIN1* in exon 1. The sequences were amplified and cloned using Q5^®^ High-Fidelity DNA Polymerase (New England Biolabs). The PCR products were cleaned with Nucleospin^®^ gel and a PCR Clean-Up Kit; (Macherey-Nagel) and then ligated into pGEM^®^-T Easy (Promega). The fragments were ligated using Golden Gate assembly in an AS orientation driven by the cauliflower mosaic virus (CaMV) *35S* promoter and terminator. To create the reporter lines, we assembled the Arabidopsis *AtPIN1* promoter, *AtPIN1*, the *GFP*-tagged histone *H2B*, and the *35S* terminator. The *Two-Component Signalling Sensor new* (*TCSn*) synthetic cytokinin-responsive promoter was fabricated by GENEWIZ^®^, Germany. The fragment was assembled to the *GFP*-tagged histone *H2B* and the *Nopaline Synthase* (*Nos*) terminator. For the auxin reporter line, the synthetic auxin-responsive *DR5* promoter was assembled to the *GFP*-tagged histone *H2B* and the *Nos* terminator. The AS constructs and the reporter lines were then inserted into a modified pBI121 vector. The constructs were then transformed into the *Escherichia coli* strain DH5α for selection. Correct constructs were then transformed into *Agrobacterium tumefaciens* strain LBA4404 by electroporation and confirmed with culture PCR.

### Transformation of *K. pinnata*

Wild type *K. pinnata* plants were transformed with *35S::KpPIN1*, *35S::KpAHP*, *35S::KpGA2ox2*, *DR5::GFP*, *AtPIN1::GFP*, and *TCSn::GFP*, as previously described (Garcěs and Sinha, 2009). The transformed *Agrobacterium* was cultivated in the dark for 48 h at 30 °C and 250 rpm on an orbital shaker in Luria Bertani (LB) medium without NaCl and supplemented with 50 mg l^–1^ rifampicin, 100 mg/ l^–1^ kanamycin, and 100 mg/ l^–1^ streptomycin. When OD_600_=0.5 was attained, the cells were centrifuged at 4000 *g* for 15 min and resuspended in liquid half-strength Murashige and Skoog (½MS) medium supplemented with 1 μl ml^–1^ of acetosyringone. Leaf fragments of 1 cm^2^ previously disinfected with 100% ethanol and commercial bleach were co-cultured with 1 ml of resuspended cells and 40 ml ½MS for 2 h in an orbital shaker in the dark, and the inoculated fragments were then cultured in the dark in MS medium without the addition of antibiotics for 2 d. The leaf fragments were then transferred to shoot-inducing medium (SIM) supplemented with 100 mg l^–1^ TDZ, 10 mg l^–1^ IAA, 50 mg l^–1^ kanamycin, and 500 mg l^–1^ carbenicillin. After 2 weeks, the tissues were transferred to SIM with 100 mg l^–1^ kanamycin and 500 mg l^–1^ carbenicillin, after which we transferred the tissues into new media every 15 d. When the leaves were formed, the explants were transferred to root-inducing medium containing ½MS media supplemented with 30 g l^–1^ sucrose and 7.5 g l^–1^ agar, and adjusted to 5.8 pH. Roots formed after 3 weeks, and the plants were then established *ex vitro* in the compost/perlite/vermiculite mix described above. Between 20–30 transgenic plants were obtained for each AS construct.

### Genotyping and phenotyping of AS lines

A quick DNA prep for PCR protocol was performed to isolate DNA (Weigel and Glazebrook, 2002). PCR was used with Q5^®^ High-Fidelity DNA and BioTaq^TM^ polymerases (Bioline, UK) as previously described (Jácome-Blásquez and Kim, 2023). The primers used were *KpPIN1*, *KpAHP*, *KpGA2ox2*, and *35S* terminator reverse primers (Supplementary Table S1). Successful incorporation of the insert was also confirmed by using forward and reverse primers to detect *NEOMYCIN PHOSPHOTRANSFERASE II* (*NPTII*) (Supplementary Table S1). The PCR settings were the ones recommended in the Q5^®^ protocol, with an annealing temperature of 56 °C and an extension time of 25 s for 35 cycles.

Leaves were excised from the mother plants, placed on a dry white paper sheet, and kept in the growth chamber under the conditions described above. New plantlet regeneration from the epiphyllous buds (EBs) was scored every 3 d up to 21 d for the WT and AS plants. New plantlets were scored when they became visible, at ∼0.5 mm in length. A total of 25 leaves (five leaves per line) were used. For measurement of crenulation depth, we used a calliper and a ruler: the ruler was placed on the two highest points of the neighbouring lobes, and we measured the distance between it and the deepest indented region. We measured five leaves per plant from each of five lines.

### Quantitative real-time PCR

Leaf crenulations of the WT, *KpPIN1*, *KpAHP*, and *KpGA2ox2* AS plants (three independent lines each) were excised and frozen in liquid nitrogen. Total RNA was extracted using RNAzol^®^ RT (Sigma-Aldrich), according to the manufacturer’s protocol. The RNA was treated with RQ1 DNase (Promega) and cDNA synthesis was achieved at 45 °C for 1 h using a Tetro cDNA Synthesis Kit (Bioline).

The quantitative real-time (RT-q)PCR reaction was prepared using 100 ng of cDNA in a 20 μl reaction containing 10 μl of SensiFAST™ SYBR Hi ROX kit (Bioline) and 1 mM of each primer 10 μM (listed in Supplementary Table S2). The reaction was performed in a StepOnePlus™ Real-Time PCR instrument with StepOne™ software v2.3. Three biological replicates were used, each with three technical repeats. *18S rRNA* was used as the control gene with an annealing temperature of 60 °C. Relative gene expression levels were calculated using the ΔΔ*C*_T_ method. A custom Microsoft Excel spreadsheet created in-house was used to compute Δ*C*_T_, ΔΔ*C*_T_, and the corresponding fold-change values.

### Statistical analysis

Data were analysed in Microsoft Excel v.16.16.27 using ANOVA followed by Dunnett’s Multiple Comparisons, and GraphPad Prism 10.1.1 was used to plot the graphs.

## Results

### Leaf morphology and plantlet formation

**Fig. 1.**
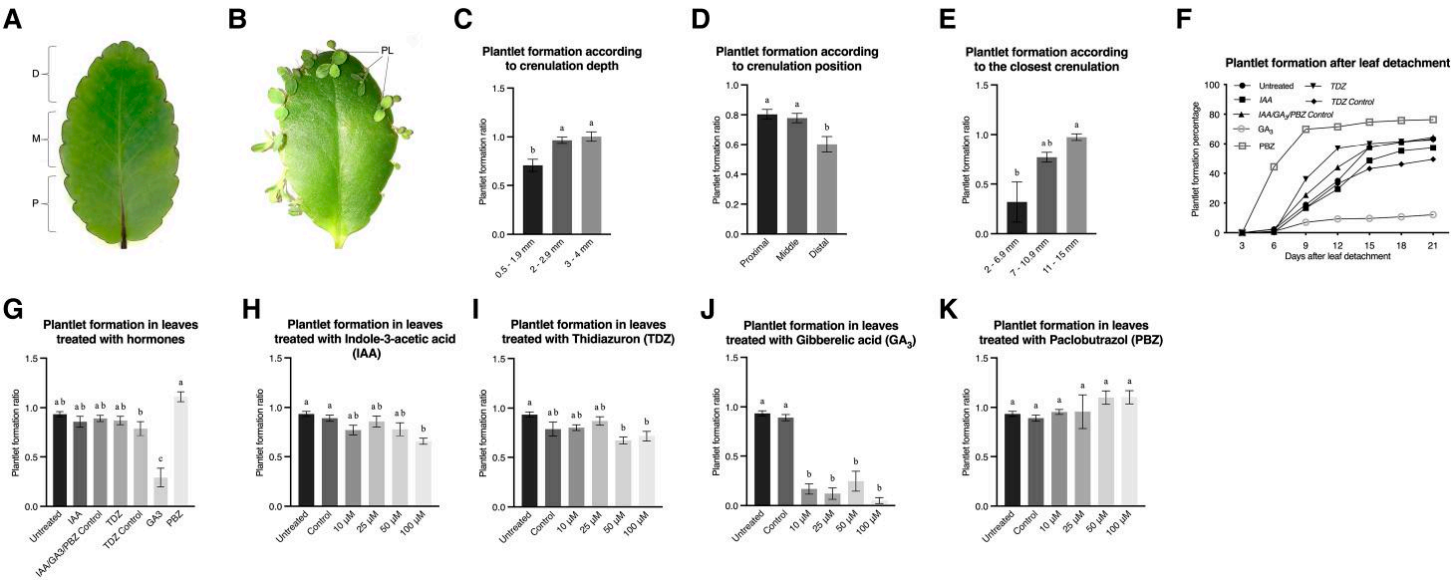
Distribution of plantlet formation in detached leaves of *K. pinnata* and effects of hormone applications. (A) Representative image indicating the proximal (P), middle (M), and distal (D) regions of the leaf. (B) Representative image of a leaf at 21 d after detachment. (C–E) Plantlet formation ratio (plantlet number/crenulation number) at 21 d after leaf detachment in relation to (C) crenulation depth, (D) the position of the crenulation in the leaf, and (E) the proximity of the crenulation to its closest neighbour. (F, G) Mature leaves were detached and treated overnight with 25 μM of IAA, GA_3_, the GA_3_ antagonist PBZ, or the synthetic cytokinin TDZ, or treated with their corresponding control solutions; ‘untreated’ indicates that no solution was applied. (F) Overall plantlet formation over 21 d after leaf detachment, expressed as a percentage of plantlet formation, and (G) overall formation at 21 d, expressed as the plantlet formation ratio. (H–K) Different concentrations of each hormone were applied overnight to the leaves: (H) IAA, (I) TDZ, (J) GA_3_, and (K) PBZ. Data are means (±SD) obtained from five leaves each from five different plants, with ∼16 crenulations per leaf. Different letters indicate significant differences among means as determined using one-way ANOVA followed by Dunnett’s multiple comparisons (*P*<0.05). The ratios were arcsine-transformed prior to statistical analysis to stabilise the variance and normalise the data.

We first investigated whether plantlet formation was influenced by leaf morphology in specific leaf crenulations. While no plantlets were formed in attached leaves, they were formed in most leaf crenulations by 21 d after detachment (Fig. 1A, B). Plantlet formation was significantly affected by the depth of leaf crenulations. with significantly more plantlets being formed in deeper crenulations (>2 mm) than in shallower ones (<1.9 mm) after 21 d of leaf detachment (Fig. 1C). In addition, plantlets mostly formed from crenulations located in the proximal region of the leaf when compared with the distal crenulations located around the tip of the leaves (Fig. 1D). Plantlets emerged almost simultaneously regardless of the position of crenulations in a leaf. Plantlet formation was affected by the proximity between crenulations, with more plantlets emerging from crenulations that >11 mm away from their closest neighbour (Fig. 1E). These results indicated that plantlet emergence is correlated with leaf crenulation.

### GA_3_ application inhibits plantlet formation

To test the effects of plant hormones on plantlet initiation, we treated detached leaves of the WT with 25 μM of IAA, TDZ, GA_3_, or the GA_3_ antagonist PBZ, and scored overall plantlet formation over the following 21 d. Plantlets started to emerge after 9 d in all the treatments except for the leaves treated with PBZ, where they emerged after 6 d (Fig. 1F). The final plantlet formation ratio (plantlet number/crenulation number) was not affected by TDZ (62%) or IAA (57.3%) in comparison with untreated leaves (63.3%; Fig. 1G); however, it was significantly decreased by GA_3_ application (12.1%) and significantly increased by PBZ (76.3%) when compared with the mock control and untreated leaves.

To investigate whether the effects of exogenous hormone applications were concentration-dependent, leaves were detached and treated with 10, 25, 50, or 100 μM of IAA, TDZ, GA_3_, and PBZ, and plantlet formation was again scored over 21 d. Leaves treated with 100 μM IAA exhibited a small but significant decrease in plantlet formation compared with untreated leaves and the control, whilst lower concentrations had no effects (Fig. 1H). Leaves treated with TDZ showed significant decreases in plantlet formation when treated with 50 μM and 100 μM (Fig. 1I). In contrast, leaves treated at all the GA_3_ concentrations consistently exhibited decreased plantlet formation compared with untreated leaves and the control (Fig. 1J). Moreover, no significant differences were observed between the different concentrations, suggesting that there was no dosage-dependent effect, and the lowest concentration was already sufficient to achieve the plateau of its action. PBZ applications tended to increase plantlet formation with increasing concentration, but the effect was not significant (Fig. 1K). Taken together, application of GA_3_ significantly reduced plantlet formation, but IAA, TDZ, and PBZ had very limited effects (Fig. 1F).

### Hormone application influences plantlet phenotypes

**Fig. 2.**
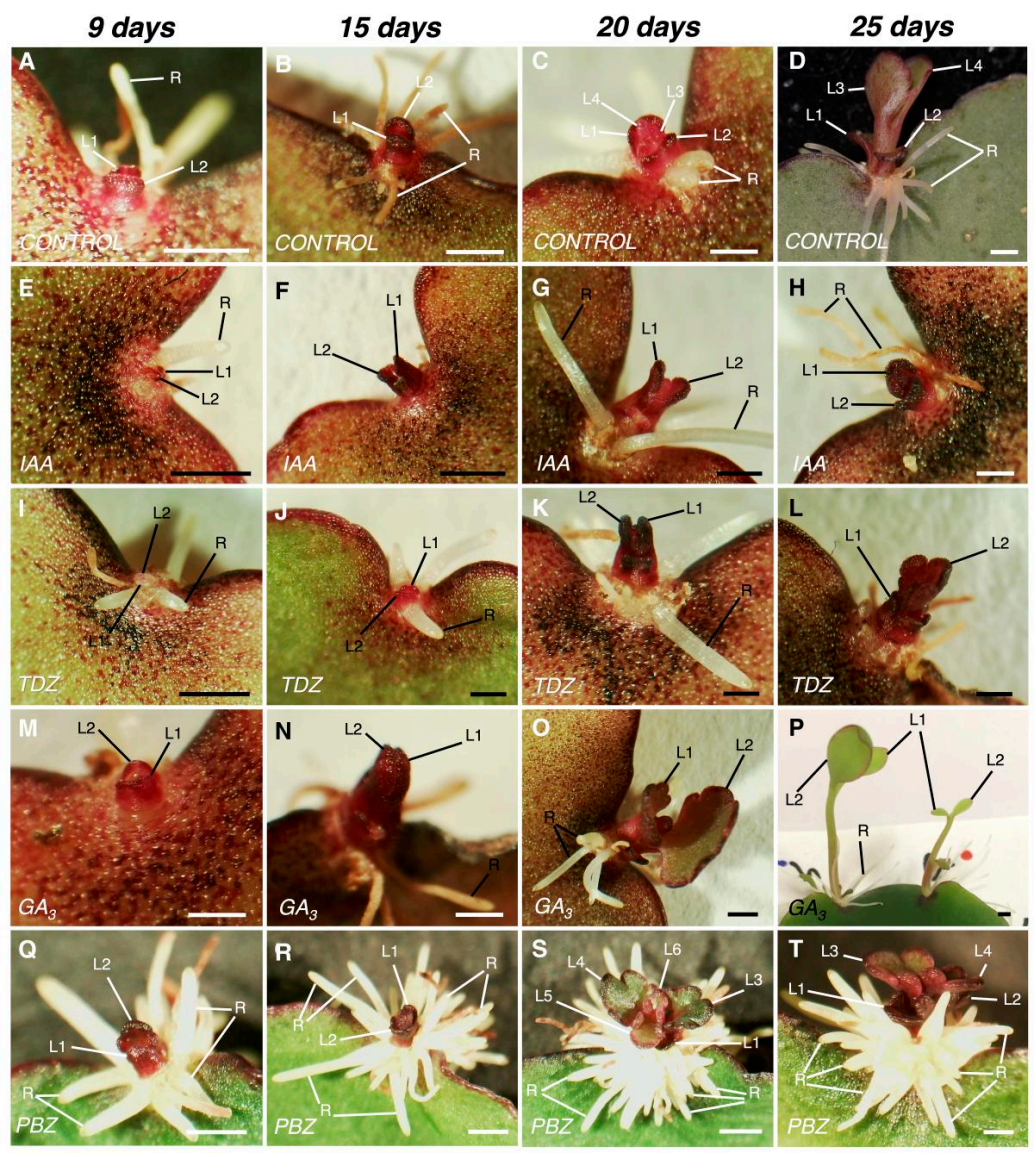
Effects of hormone applications on the induction and development of plantlets from epiphyllous buds on detached leaves of *K. pinnata*. Mature leaves were detached and treated overnight with (A–D) a control solution, or 25 μM of (E–H) IAA, (I–L) the synthetic cytokinin TDZ, (M–P) GA_3_, or (Q–T) the GA_3_ antagonist PBZ. Representative images of crenulations are shown at 9–25 d after leaf detachment, as indicated. Scale bars are 1 mm. L1–L6, leaves 1–6; R, root.

In WT leaves without hormone treatments, the first two leaf primordia (L1 and L2) of the plantlets were visible in the EBs at 9 d following leaf excision (Fig. 2A), and roots then became visible to the naked eye after 15 d (Fig. 2B). By 20 d after leaf detachment plantlets had formed leaves L3 and L4, while roots continued to develop (Fig. 2C). A fully developed plantlet at 25 d after leaf detachment is shown in Fig. 2D, with L3 and L4 having formed visible petioles and leaf crenulations. Exogenous treatment of leaves with 25 μM IAA or TDZ had little obvious effects on plantlet development and they were morphologically similar to the WT, except for delayed L3 and L4 development (Fig. 2E–L). GA_3_ treatment showed inhibitory effects on plantlet formation, but a few were able to emerge. Initially, the emerging plantlets were similar to those of the WT at 9 d after leaf detachment (Fig. 2M); however, the subsequent developing plantlet showed slightly elongated L1 and L2 at 15 d (Fig. 2N). After 20 d, the leaves of the plantlets were bigger than those observed in untreated leaves (Fig. 2O), and 25-day-old plantlets showed an elongated stem and an underdeveloped root system (Fig. 2P). Conversely, leaves treated with 25 μM PBZ had plantlets with over-proliferation of the roots and shorter internodes of the stem. In this treatment, plantlets emerged earlier and were visible from 6 d after detachment. By 9 d, their leaves were substantially bigger than those of the WT, and numerous short roots were present (Fig. 2Q). The roots continued to proliferate from 15–25 d (Fig. 2R–T); however, the leaves of plantlets looked similar to those of the at 20 d and 25 d (Fig. 2S, T). Thus, exogenous hormone application affected not only plantlet formation but also their phenotypes. Notably, although the hormone treatments were applied only once after the leaf detachment, they affected both the initial and subsequent development of the plantlets.

### Phenotypes of *KpPIN1*, *KpAHP* and *KpGA2ox2* antisense lines

To further confirm the roles of hormones in plantlet formation, we generated transgenic lines with overexpressing AS fragments of *KpPIN1*, *KpAHP*, and *KpGA2ox2* under the cauliflower mosaic virus *35S* promoter. At least six independent lines per gene were obtained and were PCR-confirmed by amplifying the transgenes and *NPTII* gene (Supplementary Table S1; Supplementary Fig. S1). Successful down-regulation of the genes was confirmed by RT-qPCR (Supplementary Fig. S2A, E, I).

**Fig. 3.**
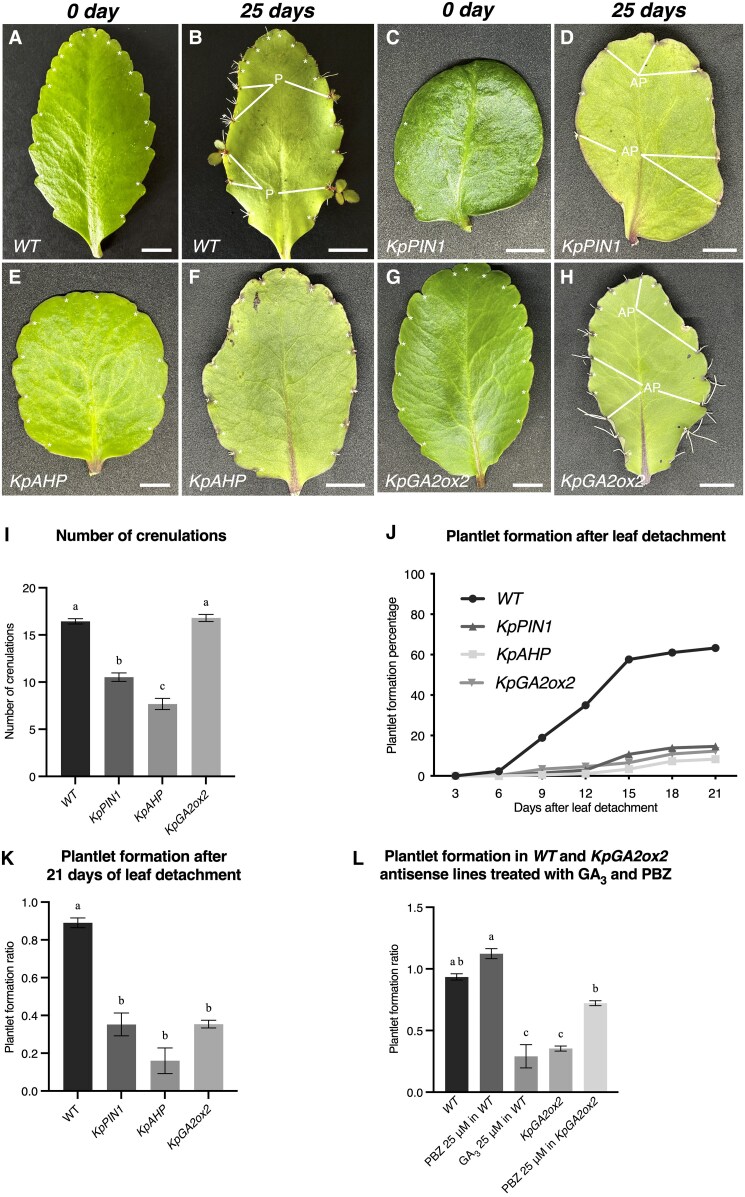
Comparison of leaf phenotypes between the *K. pinnata* wild type (WT) and transgenic antisense (AS) lines for *KpPIN1*, *KpAHP*, and *KpGA2ox2*. (A–H) Representative images of leaves of (A, B) the WT, (C, D) *KpPIN1*, (E, F) *KpAHP*, and (G, H) *KpGA2ox2* immediately following leaf detachment (0 d) or 21 d later, as indicated. *, leaf crenulation; AP, aborted plantlet; P, plantlet. Scale bars are 1 cm. (I) Number of crenulations per leaf in AS plants. (J) Percentage of crenulations with plantlet formation from 0–21 d after leaf detachment. (K) Plantlet formation ratio (plantlet number/crenulation number) at 21 d after leaf detachment. (L) Detached leaves of the WT and the *KpGA2ox2* AS lines were treated overnight with 25 μM of GA_3_ or the GA_3_ antagonist PBZ and the plantlet formation ratio was determined after 21 d. Data are means (±SD) obtained from five leaves each from five different replicate plants. Different letters indicate significant differences among means as determined using one-way ANOVA followed by Dunnett’s multiple comparisons (*P*<0.05). The ratios were arcsine-transformed prior to statistical analysis to stabilise the variance and normalise the data.

Mature WT leaves exhibited left–right symmetry and formed an average of 18 crenulations with a number of plantlets emerging by 25 d following detachment (Fig. 3A, B, I). Mature leaves of the *KpPIN1* AS lines exhibited a thicker appearance than WT leaves, and displayed strong asymmetry and a rounded shape (Fig. 3C, D). They did not present any plantlet formation at 25 d; however, some crenulations exhibited root initiation before undergoing abortion (Fig. 3D, ‘AP’). Notably, the crenulations on these leaves were either extremely shallow or absent (Fig. 3C, D). The *KpPIN1* AS lines formed leaves with significantly fewer crenulations compared with the WT (Fig. 3I). Similar to the *KpPIN1* lines, the leaves of the *KpAHP* AS lines were rounder and had less left–right symmetry than the WT, and they also had shallower crenulations with a shorter proximal–distal distance (Fig. 3E, F). The leaves of the *KpAHP* AS plants had significantly fewer crenulations compared with the WT (Fig. 3I) and had no visible plantlet formation 25 d after leaf detachment (Fig. 3F). The *KpGA2ox2* AS lines developed WT-like leaves in terms of shape and number of crenulations, except that the lobes of crenulation were rounder than those of the WT (Fig. 3G–I,). Similar to the *KpAHP* lines, the leaves of the *KpGA2ox2* AS lines had fewer plantlets at 25 d after leaf detachment and the developing EBs presented long roots but showed almost no leaf development (Fig. 3H). These results showed that down-regulating *PIN1* and *AHP* affected the leaf phenotypes, whereas down-regulation of *KpGAox2* did not alter leaf morphology. More importantly, all the AS lines formed fewer plantlets than the WT.

We next examined plantlet formation for 21 d following leaf excision, and found that it was significantly delayed and disrupted in all the AS lines (Fig. 3J). In WT leaves, plantlets started to form from day 9 and continued to day 15, whereas in the AS lines this initial active plantlet formation was absent. At 21 d after detaching the leaves, the plantlet formation percentage was 63.3% for the WT, 14.5% for *KpPIN1*, 8.3% for *KpAHP*, and 12.3% for *KpGA2ox2* (Fig. 3K). All the AS lines formed significantly fewer plantlets than the WT.

We next investigated whether the GA_3_ antagonist PBZ was able to rescue the *KpGA2ox2* AS phenotype. Following treatment of leaves with 25 μM PBZ, we found that plantlet formation exhibited a slight increase in the WT treated, but a significant increase was observed in *KpGA2ox2* compared with untreated *KpGA2ox2* plants (Fig. 3L). Additionally, it was notable that the reduced plantlet formation in GA_3_-treated WT leaves was comparable with that in the *KpGA2ox2* AS lines. This suggested that the depletion of GA_3_ from the leaves of the *KpGAox2* lines induced plantlet emergence.

### Down-regulation of *KpPIN1*, *KpAHP*, and *KpGA2ox2* affects plantlet phenotypes

**Fig. 4.**
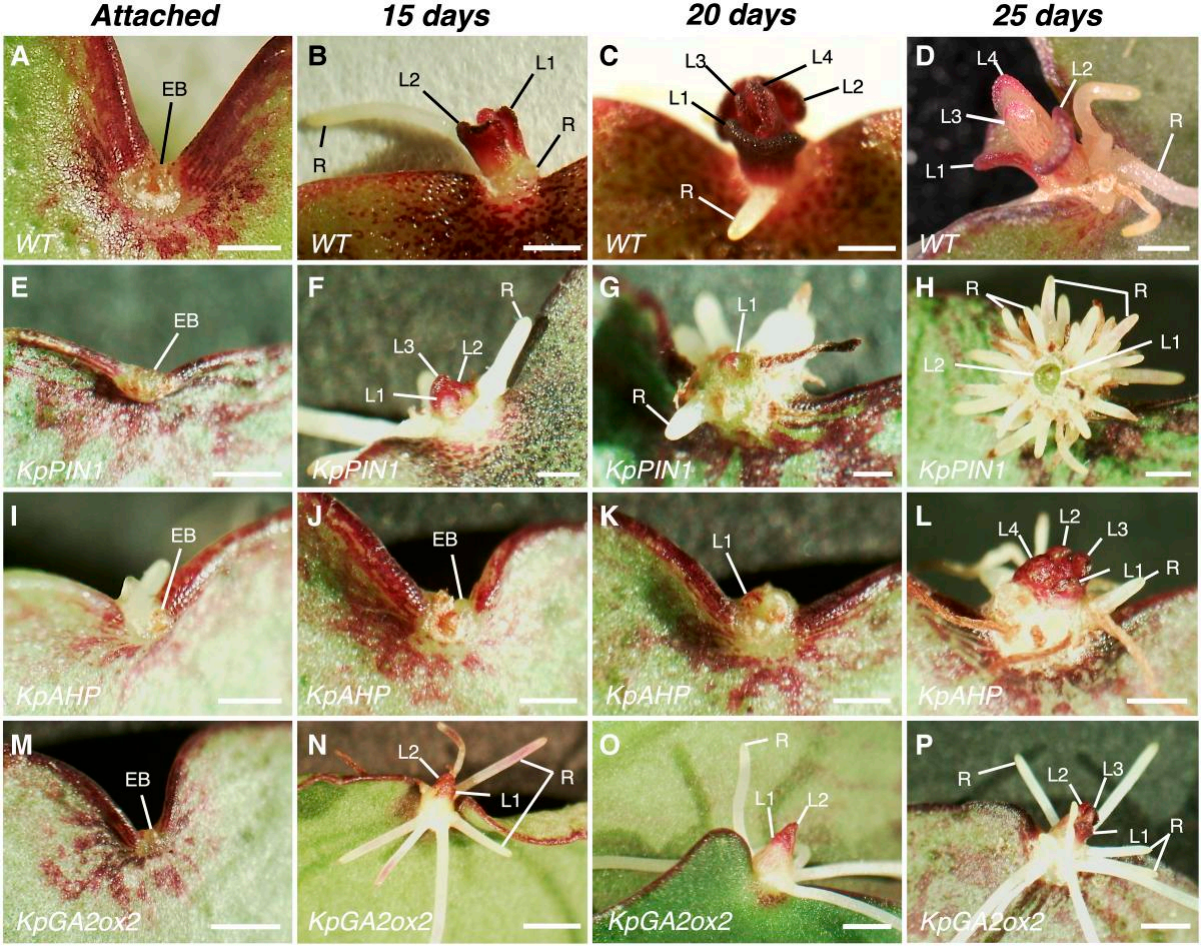
Plantlet phenotypes of the *K. pinnata* wild type (WT) and transgenic antisense (AS) lines for *KpPIN1*, *KpAHP*, and *KpGA2ox2*. Representative images of leaf crenulations in (A–D) the WT, (E–H) *KpPIN1*, (I–L) *KpAHP*, and (M–P) *KpGA2ox2* in attached leaves and at 15–25 d after leaf detachment, as indicated. Scale bars are 1 mm. EB, epiphyllous bud; L1–L4, leaves 1–4; R, root.

Attached, mature leaves of the WT showed EBs in the middle of the leaf crenulations (Fig. 4A). At 15 days after leaf detachment, the plantlet leaves L1 and L2 were visible, and roots had emerged and elongated, whilst after 20 d leaves L3 and L4 had formed, and further root growth had occurred (Fig. 4B, C; see also Fig. 2). After 25 d, the L3 and L4 leaves had developed and formed leaf crenulations (Fig. 4D). In the *KpPIN1* AS plants, the EBs were also evident in the middle of the shallow crenulations in attached leaves (Fig. 4E), and at 15 d after detachment, L1, L2, and L3 together with roots had emerged (Fig. 4F). However, the leaves did not develop whilst the roots had over-proliferated at 20 d and 25 d (Fig. 4G, H). Attached leaves of *KpAHP* AS plants showed WT-like EBs (Fig. 4I). At 15 d and 20 d after leaf detachment, only slow plantlet initiation with no root initiation could be seen (Fig. 4J, K). The EBs increased in size similar to the WT; however, there was no clear plantlet morphogenesis, and neither leaves nor roots emerged. At 25 d after leaf detachment, the *KpAHP* AS plantlets showed underdeveloped L1, L2, L3, and L4 leaves (all four primordia emerged at the same time) that stayed in a scale-like form, together with some roots (Fig. 4L). Attached leaves of the *KpGA2ox2* AS lines also had EBs similar to those of the WT (Fig. 4M). At 15 d following leaf detachment, the EBs developed plantlets showing L1 and L2 and numerous roots (Fig. 4N). The plantlets continued to develop long roots; however, the leaves showed slower growth in comparison with WT after 20 d (Fig. 4O), so that 25-day-old plantlets showed underdeveloped leaves and over-proliferation of roots, which were both more numerous and longer than those of the WT (Fig. 4P). These results indicated that the plantlet phenotypes were altered by the down-regulation of the hormone genes; all the AS lines formed plantlets with underdeveloped leaves, and *KpPIN1* and *KpGA2ox2* exhibited root over-proliferation.

### Expression of *KpPIN1*, *KpAHP*, *KpGA2ox2*, and *KpYUC1* in the antisense lines

We next used RT-qPCR analysis to determine whether the down-regulation of the individual transgenes affected the expression of other hormone-related genes, and the results are shown in Supplementary Fig. S2. Analysis of the crenulation area in attached mature leaves of the AS lines indicated the successful down-regulation of the target genes in each of these lines (Supplementary Fig. S2A, E, I). We found that *KpYUC1*, *KpPIN1*, and *KpGA2ox2* were significantly down-regulated in all the *KpAHP* AS lines. *KpAHP* and *KpPIN1* were down-regulated in all the *KpGA2ox2* AS lines, but *KpYUC1* expression was not affected. *KpYUC1* was down-regulated in all three *KpPIN1* lines whilst, *KpGA2ox2* was down-regulated in two of the lines and *KpAHP* expression was not affected in any of the *KpPIN1* AS lines. This showed that the expression level of one hormone gene could affect the expression of other hormone-related genes.

### *DR5*, *PIN1*, and *TCSn* expression in leaf crenulations correlates with plantlet formation

**Fig. 5.**
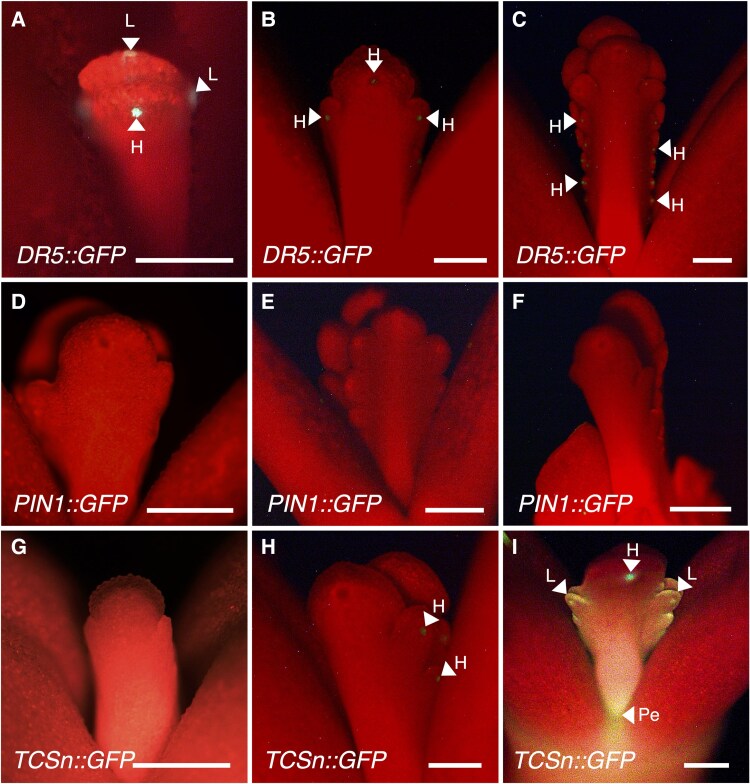
Spatial distribution of auxin and cytokinin in emerging leaves of the shoot apical meristem of *K. pinnata*. The promoters of the synthetic auxin-responsive *DR5*, *PIN1*, and the synthetic cytokinin-responsive *Two-Component Signalling Sensor new* (*TCSn*) were used to drive *GFP* expression. Representative images are shown of the GFP signals in (A–C) *DR5*::*GFP*, (D–F) *PIN1*::*GFP*, and (G–I) *TCSn::GFP* Scale bars are 5 mm. Arrowheads indicate the presence of GFP signals in leaf lobes (L), hydathodes (H), and petioles (Pe).

**Fig. 6.**
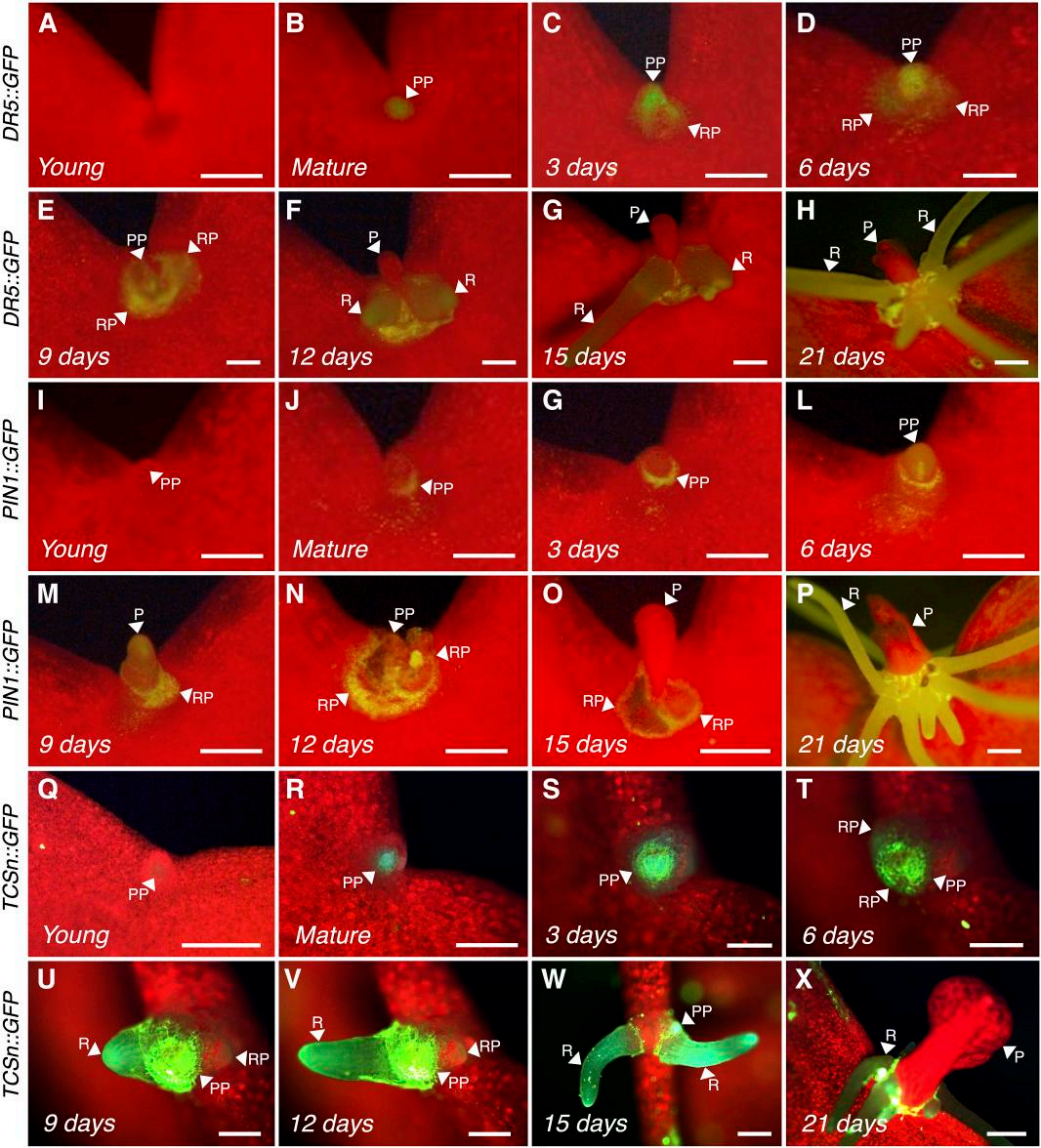
Spatial distribution of auxin and cytokinin in developing plantlets on detached leaves of *K. pinnata*. The promoters of the synthetic auxin-responsive *DR5*, *PIN1*, and the synthetic cytokinin-responsive *Two-Component Signalling Sensor new* (*TCSn*) were used to drive *GFP* expression. Representative images of the GFP signals in (A–H) *DR5*::*GFP*, (I–P) *PIN1*::*GFP*, and (Q–X) *TCSn::GFP* plants are shown. Epiphyllous buds (EBs) in young, developing leaves of each genotype are presented in (A), (I), and (Q), and in attached mature leaves in (B), (J), and (R). Leaves were then detached, and the number of days after detachment is indicated in each image. Scale bars are 5 mm. P, plantlet; PP, plantlet primordium; R, root; RP, root primordium.

To investigate the spatial distribution of auxin and cytokinin during plantlet formation, we generated transcriptional reporter lines using GFP. The synthetic auxin-responsive promoter *DR5*, the promoter of the *AtPIN1* auxin efflux carrier, and the *TCSn* synthetic cytokinin-responsive promoter were used to drive the expression of *GFP*. We first examined attached emerging leaves of the shoot apical meristem (SAM) and found that the GFP signal in the *DR5::GFP* lines was initially detected in the emerging lobes and hydathodes (Fig. 5A). Later, the signal was seen only in the hydathodes of each lobe (Fig. 5B). As the first crenulations were developed on the emerging leaves the GFP signal faded from the largest, distal hydathodes and became visible only in the younger, growing ones. No GFP signal was detected in newly emerged and developing leaves of the *PIN1::GFP* line (Fig. 5D–F). Similarly, no signals were detected in new leaves emerging from the SAM in the cytokinin *TCSn::GFP* lines (Fig. 5G). However, signals were evident later in the hydathodes (Fig. 5H), in then the leaf lobe margins, in the petiole, and in leaves with developing crenulations (Fig. 5I). No signal was detected within the indentation of the crenulations (the sinus region) during the early stages of leaf development in any of the reporter lines. During later stages of development of attached leaves (2–3 cm long), the GFP signal was not visible in either the *DR5*::*GFP* or *PIN1::GFP* lines (Fig. 6A, I); however, it was evident in the sinus of the *TCSn::GFP* lines (Fig. 6Q). In mature, attached leaves, the GFP signal was detected in EBs in all three reporter lines (Fig. 6B, J, R); it was in the centre of the EB primordium dome in *DR5::GFP* and *TCSn::GFP* lines (Fig. 6B, R) but in the peripheral and basal region of the EB dome in *PIN1::GFP* (Fig. 6J).

We also investigated GFP signals after the mature leaves were detached. At 3–9 days after detachment, the signal intensified in all three lines (Fig. 6C–E, K–M, S–U). In particular, the signal extended to the centre of the EB dome in *PIN1::GFP* leaves after 6–9 d (Fig. 6L, M). The signal was detected in emerging roots in all three linesfrom 9 d after detachment onwards (Fig. 6E–H, M–P, U–X). The GFP signal was visible in the entire roots of the *DR5::GFP* and *TCSn::GFP* lines but only in the base of the emerging roots in the *PIN1::GFP* lines (Fig. 6N, O). No obvious GFP signal was detected in emerging plantlet leaves of any of the three lines (Fig. 6G, H, N–P, V–X). In summary, the reporter lines showed that auxin and cytokinin accumulation coincided with the formation of EBs in the sinus region of mature leaves, and the accumulation intensified with the initiation of plantlet formation after leaf detachment.

## Discussion

Epiphyllous buds (EBs) are the primordia located in the leaf margins of inducible plantlet-forming *Kalanchoë* species, and they remain dormant until leaf detachment triggers plantlet initiation. It has been proposed that signals travelling through leaf veins to the EBs prevent plantlet formation until they are disrupted by leaf excision, thereby releasing plantlet dormancy (Naylor, 1932; Sawhney *et al*., 1994). This suggests the presence of a correlative inhibition process, comparable to that observed in apical dominance (Sawhney *et al*., 1994) where auxin is transported from the SAM to the stem, inhibiting axillary and lateral bud development (Dun *et al*., 2006). Conversely, cytokinin is usually transported from the roots to the shoot through the xylem, promoting axillary and lateral growth (Pearce *et al*., 1995). Our external hormone treatments suggested that breaking the dormancy of EBs in *K. pinnata* appears to be different from apical dormancy. Auxin and cytokinin applications did not affect plantlet formation (Fig. 1). However, the depth and distance to the neighbouring crenulation as well as the proximo-distal position of the crenulation affected EB formation significantly, suggesting that innate hormonal distributions might be important for EB formation.

A wide variety of dormancy-breaking responses of the primordia following the external application of hormones has been observed within the *Kalanchoë* genus. Plantlet formation in *K. daigremontiana* is normally induced under long-day conditions, but application of the cytokinin 6-benzylamine-purine (BAP) induces formation under short-day conditions (Heide, 1965). The same study also showed that attached leaves of *K. pinnata* produce plantlets without roots after being treated with BAP. Consistent with this, leaf explants of *K. pinnata* with EBs cultured *in vitro* in media supplemented with TDZ grow into dwarf and rootless plantlets (Jaiswal and Sawhney, 2006), suggesting exogenous cytokinin applications inhibit root development. However, in *K. marnieriana*, the cytokinins zeatin, kinetin, and BAP inhibit plantlet formation, whereas the cytokinin antagonist purine riboside promotes it (Kulka, 2006). In contrast, our results suggested that external application of cytokinin did not affect plantlet formation in detached leaves of *K. pinnata* or the plantlet morphology (Figs 1, 2L), although it is important to note that our treatments were applied only once to the leaves and were not maintained throughout plantlet development.

Low doses of IAA stimulate plantlet formation in leaf explants of *K. daigremontiana* cultured *in vitro*, but the opposite effect is observed when it is applied to attached leaves (Yazgan and Vardar, 1977). An auxin 1-naphthaleneacetic acid (NAA) inhibits plantlet formation and terminal and axillary bud development in *K. daigremontiana* and *K. pinnata* (Heide, 1965). Furthermore, it has been demonstrated that within developing plantlets of *K. marnieriana*, auxin synthesised in the SAMs is transported to promote root formation (Kulka, 2008). In our study, external application of IAA showed no significant effect on the initiation of plantlet primordia of *K. pinnata*; however, a slight decrease in the plantlet formation ratio was observed when compared with the controls (Fig. 1). The plantlet morphology was not affected after the IAA treatment (Fig. 2). These results suggest that it is likely that auxin does not participate in the break of dormancy of the EBs that follows the detachment of the leaves, or at least that the concentrations we used were not sufficient to trigger any response in *K. pinnata*.

External application of GA_3_ inhibited plantlet formation in detached *K. pinnata* leaves, whereas the GA_3_ antagonist PBZ promoted plantlet formation and early plantlet emergence (Fig. 1F). This suggested that GA pathways might be involved in breaking the dormancy in the EBs of *K. pinnata*, with the supply of GA_3_ to attached leaves inhibiting plantlet formation. Reduced GA_3_ activity promotes meristematic activity through *STM* expression in the SAM of Arabidopsis (Hay *et al*., 2002), and it has also been shown that plantlet-forming *Kalanchoë* species express the *STM* ortholog in the plantlet primordia together with other meristem genes to facilitate plantlet formation (Garces *et al*., 2007; Jácome-Blásquez and Kim, 2023). This suggests that plantlet formation in the *Kalanchoë* genus might have commenced with the ectopic expression of meristem genes in the leaf margins. Thus, it might be possible that the GA pathway interacts with the meristem pathways during EB dormancy. This could be investigated by examining the expression of GA and meristem genes such as *STM* and *WUS* in the EBs of transgenic GA or meristem antisense plants.

To investigate the molecular regulation of hormones in plantlet formation in *K. pinnata*, we generated AS lines with down-regulation of *KpPIN1*, *KpAHP*, and *KpGA2ox2*. In Arabidopsis, *AHP6* mediates the crosstalk between cytokinin and auxin to control the rate of organ initiation from the SAM (Besnard *et al*., 2014). Additionally, Arabidopsis mutants with defective cytokinin pathways show fewer leaf serrations than the WT (Navarro-Cartagena and Micol, 2023). Consistent with this, the *KpAHP* AS lines had significantly fewer crenulations in the leaves when compared with the WT (Fig. 3). In addition, we observed that plantlet formation was severely disrupted in the leaves of these lines. This suggested that cytokinin pathways mediated the formation of the leaf crenulations (and thus the formation of EBs) and facilitated plantlet initiation and development. *AHP6* in Arabidopsis also provides orientation for cell division during lateral root formation and influences the localisation of *PIN1* during their initiation (Moreira *et al*., 2013). Our expression analyses showed that *KpPIN1* was significantly down-regulated in the *KpAHP* AS lines (Supplementary Fig. S2C), suggesting that *KpAHP* also participates in auxin transport and adventitious root formation during plantlet initiation in *K. pinnata*. The plantlets that formed from the EBs of the *KpAHP* AS lines showed leaf deformities, with the first and second pairs of leaf primordia emerging late and at the same time (Fig. 4I–L). In Arabidopsis, *AHP6* expresses in the SAM and its protein (which inhibits cytokinin signalling) moves to the organ primordia and generates a concentration gradient between the two earliest initiated organs that promotes sequential organogenesis (Besnard *et al*., 2014). Our results indicated that the *KpAHP* ortholog might be mediating the progression of organogenesis in the plantlets of *K. pinnata*.

*PIN1* and *CUC2* maintain feedback loops in the leaf margins of Arabidopsis to form serration patterns (Bilsborough *et al*., 2011). We have previously shown that AS lines of *K. pinnata* with low expression of *KpCUC2* produce leaves with shallow crenulations with significantly fewer leaf notches than the WT (Jácome-Blásquez and Kim, 2023). Similarly, the leaves of *KpPIN1* AS lines had significantly fewer crenulations (Fig. 3), confirming that auxin efflux to the leaf margins facilitated by *KpPIN1* specifies the crenulations during leaf development. Plantlet formation in the *KpPIN1* AS lines was strongly disrupted as fewer crenulations led to fewer EBs. The occasional plantlets that were formed had underdeveloped leaves and over-proliferation of roots (Fig. 4E–H), confirming the role of auxin in leaf and root development. This highlights that auxin efflux is essential for SAM development in the plantlets, and that the functions of other *KpPIN* genes do not overlap with that of *KpPIN1*.

Unlike *KpAHP* and *KpPIN1*, the *KpGA2ox2* AS lines showed WT-like leaves and the number of crenulations and the EBs were not affected (Figs 3, 4M), suggesting that their formation was not influenced by GA pathways. However, the external GA_3_ application strongly inhibited plantlet formation in the WT whilst PBZ resulted in premature initiation and tended to increase in the number of plantlets (Fig. 1). This suggested that depletion of GA_3_ from leaves of *K. pinnata* was crucial for plantlet initiation. Endogenous bioactive GA_3_ levels are regulated by the balance of its biosynthesis and deactivation (Yamaguchi, 2008). Deactivation is catalysed by GA_2_ oxidases via 2β-hydroxylation (Li *et al*., 2019), which is the main pathway to regulate bioactive GA_3_ in Arabidopsis (Rieu *et al*., 2008). Seeds of Arabidopsis *GA2ox2* loss-of-function mutants fail to germinate due to the accumulation of gibberellin A4 (GA_4_) (Yamauchi *et al*., 2007). Similarly, *KpGA2ox2* AS lines presented delayed plantlet formation (Fig. 3J), and some phenotypes showed severe disruption of plantlet formation. We also observed that external PBZ application to the leaves of *KpGA2ox2* AS lines restored plantlet formation (Fig. 3L). This confirms that the presence of GA_3_ in the leaves of *K. pinnata* maintains the dormancy of EB, thereby inhibiting plantlet formation. Our results also suggest that the main pathway to deactivate GA_3_ in the leaves of *K. pinnata* is mediated by the GA_3_ oxidase *GA2ox2*.

We also found that the down-regulation of *KpPIN1* led to down-regulation of *KpYUC1* and *KpGA2ox2* (Supplementary Fig. S2). This agrees with the previous finding in Arabidopsis that auxin modulates genes involved in GA metabolism, such as *GA2ox* (Frigerio *et al*., 2006). It also suggests that the phenotypes we observed in the *KpPIN1* transgenics might not be entirely and directly due to the down-regulation of *PIN1*, as they could be due to the decreases in expression of *KpYUC1* or *KpGA2ox2*. Auxin distribution models in Arabidopsis have shown that shoot induction is orchestrated by auxin gradients oriented away from the sites of biosynthesis by PIN1 (Abley *et al*., 2016). Our results suggested that the down-regulation of the auxin transporter *KpPIN1* also affected auxin biosynthesis in the leaf crenulations. However, it is also possible that auxin synthesis modulated by *KpYUC1* was low in the crenulation area and that the auxin that was present was mostly transported into this area by KpPIN1. In addition, the down-regulation of *KpGA2ox2* in the AS lines resulted in a down-regulation of *KpAHP* and *KpPIN1* (Supplementary Fig. S2), once again suggesting that there are interactions between the auxin, gibberellin, and cytokinin pathways during leaf development and plantlet formation in *K. pinnata*.

The auxin and cytokinin reporter lines showed GFP signals in newly emerging leaves in the shoot (Fig. 5). The signals appeared as the leaves developed and indicated the accumulation of these two hormones at the flanks of the emerging leaves, possibly specifying where the crenulations will form. In Arabidopsis, auxin is transported to the leaf margins by PIN1, where it interacts with *CUC2* to determine the leaf serrations (Bilsborough *et al*., 2011), and the leaf crenulations of *K. pinnata* might also be determined by auxin accumulation and *KpCUC2* expression. In Arabidopsis, *STM* and *CUC2* modulate each other during SAM development (Scofield *et al*., 2018) and the *Kalanchoë STM* ortholog has been reported to be ectopically expressed in the leaf margins of plantlet-forming species (Garces *et al*., 2007; Jácome-Blásquez and Kim, 2023). In addition, in a previous study we have shown that meristem genes are expressed in the centre of the EBs of mature leaves (Jácome-Blásquez and Kim, 2023), where the *TCSn*::*GFP* and *DR5*::*GFP* signals were also visible (Fig. 6). This provides further evidence that auxin and cytokinin might act synergically to establish the leaf crenulations during leaf development and possibly facilitate the expression of meristem genes to form the EBs. Our GFP lines also showed a strong accumulation of signals in plantlet primordia after leaf detachment, suggesting that auxin and cytokinin are involved in plantlet development after EB dormancy is broken by a drop in GA_3_ following the detachment. This also indicates that auxin transport to the EBs is required during plantlet formation. Our results point towards auxin transport and accumulation at the leaf margin driving the formation of the leaf crenulations and supporting the formation of plantlets after leaf excision.

### A model of plantlet formation

**Fig. 7.**
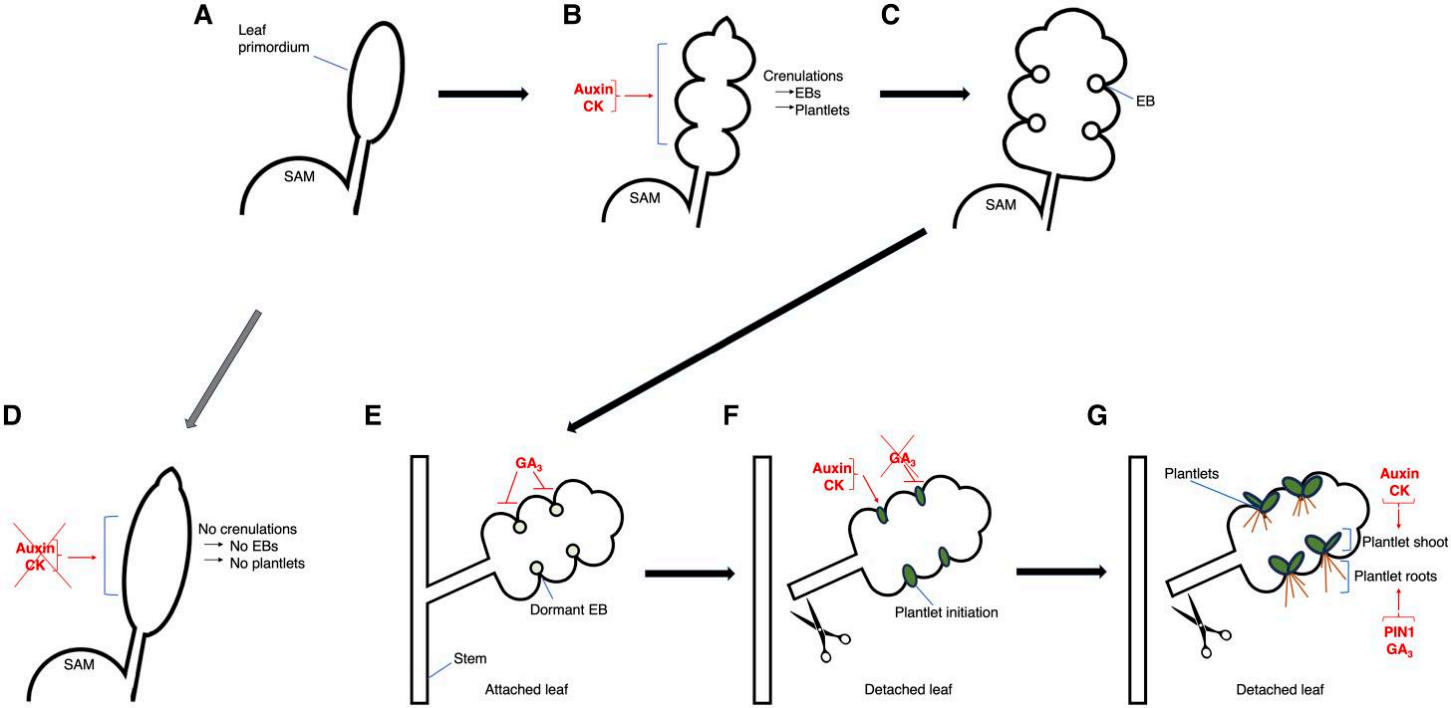
A schematic model for plantlet formation and hormones. Auxin and cytokinin (CK) are involved in leaf crenulation, which is required for epiphyllous bud (EB) formation (A–C). Absence of auxin and CK results in loss of crenulation, leading to no EBs (D). In an attached leaf, GA_3_ inhibits plantlet initiation (E), but once the leaf is detached, GA_3_ is depleted and auxin and CK accumulation promote plantlet initiation (F). Later, all three hormones cooperate to promote plantlet development (G).

Based on our findings, we propose the following working model. During early leaf development, auxin and cytokinin (but not GA_3_) are required for the establishment of leaf crenulations, where EBs will be formed during later stages (Fig. 7A–C). The loss of crenulations results in the absence of EBs, consequently preventing the formation of plantlets (Fig. 7D). GA_3_ (not auxin or cytokinin) inhibits plantlet initiation and keeps EBs dormant in mature attached leaves (Fig. 7E). Upon leaf detachment, GA_3_ depletion and accumulation of auxin and cytokinin in EBs promote plantlet initiation (Fig. 7F). After plantlet initiation, auxin, cytokinin, and GA_3_ regulate plantlet development through an interactive feedback network (Fig. 7G).

## Conclusion

Our study highlights the complementary roles of auxin and cytokinin in the processes of leaf formation in *K. pinnata*, including the development of leaf crenulations and the establishment of undifferentiated cells essential to form the epiphyllous buds. Moreover, our findings emphasise the function of GA_3_ pathways in releasing dormancy in these buds to initiate plantlet formation. This study provides insights into the complex molecular mechanisms and regulatory networks that orchestrate asexual vegetative reproduction in *K. pinnata*.

## Supplementary Material

eraf405_Supplementary_Data

## Data Availability

The sequences of the fragments used to create the AS lines have been deposited in GenBank under the following accession numbers: *KpPIN1*, PP700249; *KpAHP*, PP683461; and *KpGA2ox2*, PP700250.

## Supplementary data

The following supplementary data are available at *JXB* online.

Table S1. List of primers for gene cloning and genotyping.

Table S2. List of primers used for RT-qPCR.

Fig. S1. Genotyping of the antisense lines.

Fig. S2. Expression analysis of *KpPIN1*, *KpAHP*, *KpGA2ox2*, and *KpYUC1* in the three antisense lines.
