# Supplementary material for: Plant hormones regulate inducible asexual reproduction in Kalanchoë pinnata
Source: J Exp Bot. 2025 Sep 16;76(22):6896–910. doi: 10.1093/jxb/eraf405 (PMC12675274; doi:10.1093/jxb/eraf405)
Supplement: eraf405_Supplementary_Data [file eraf405_supplementary_data.pdf]

Authors: Francisco Jácome-Blásquez[1, 2], Joo Phin Ooi[1], Itzel M. Viveros-Sánchez[1], Victoria Spencer[1, 3] Yiğit Berkay Gündoğmuş[1] and Minsung Kim[1*]

[*]**Corresponding author:** Minsung Kim/ Telephone +44 (0) 161 275 1575; email minsung.kim@manchester.ac.uk; ORCID 0000-0002-8470-793X

[1] Division of Molecular and Cellular Function, School of Biological Sciences, Faculty of Biology, Medicine and Health, The University of Manchester, Manchester, M13 9PT, United Kingdom

[2] Current Affiliation: Apollo, OPEN Health Communications, Manchester, M1 5AN, United Kingdom

[3] Current Affiliation: Gregor Mendel Institute of Molecular Plant Biology, Austrian Academy of Sciences, Bohr-Gasse 3, 1030 Vienna, Austria

Co-authors' email addresses and ORCID:

Francisco Jácome-Blásquez - Francisco.jacomeblasquez@outlook.com - 0000-0002-6340-434X

Joo Phin Ooi - joophin.ooi@outlook.com - 0009-0001-9302-548X

Itzel M. Viveros-Sánchez - itzelmargarita.viverossanchez@postgrad.manchester.ac.uk - 0000-0002-0340-2537

Victoria Spencer - victoria.spencer@gmi.oeaw.ac.at - 0000-0002-9930-1377

Yiğit Berkay Gündoğmuş - yigit.gundogmus@manchester.ac.uk - 0000-0001-9806-7497

Running title: Regulation of asexual reproduction in *K. pinnata* by plant hormones

Supplementary data: 2 tables, 2 figures.

Table S1. Primers for gene cloning and genotyping

Module	Insert		Primer 5’→ 3’	Size
p35S	*35S CaMV* promoter	Forw	gtggtctca **GGAG** GCTAGAGCAGCTTGCCAAC	833
		Rev	gtggtctca **CACC** GGTCGATCGACAGATCTGCG	
KpAHP	*K. pinnata AHP* exon 2 (AS)	Forw	gtggtctct **AAGC**CGACTACACCAACTCCCTC	223
		Rev	gtggtctct **GGTG**GCTGCTGCCCTTCAACTG	
KpGA2ox2	*K. pinnata GA2ox2* exon 1 (AS)	Forw	gtggtctct **AAGC**CATTAGAAACCACCTTCATC	338
		Rev	gtggtctct**GGTG**GGCGAAGAGTAAATACTCG	
KpPIN1	*K. pinnata PIN1* exon 1 (AS)	Forw	gtggtctct **AAGC**CTCCACCAACAACCCCTAC	171
		Rev	gtggtctct **GGTG**GGAGACGGAGAAGAGAGTG	
35S Term	*35S CaMV* Terminator	Forw	gtggtctct **GCTT** GGGACTCTGGGGTTCGGATC	237
		Rev	gtggtctct**AGCG** GGTGATCTGGATTTTAGTACTGG	
NPTII	*NPTII*	Forw	CACAACAGACAATCGGCTGC	
		Rev	GCACGAAGCGGTCAG3	

Table S2. List of primers used for RT-qPCR

Target	Primer Name	Sequence 5'→ 3'
qKpAHP	*qKpAHP* Forw	GTTTTCCCAGTCACGAC
	qKpAHP Rev	CAGGAAACAGCTATGAC
qKpPIN1	*qKpPIN1* Forw	ACAGTCAGGAAGTCCAACGC
	qKpPIN1 Rev	TGCATTCGTGAGGTTGGAGG
qKpGA2ox2	qKpGA2ox2 Forw	TCAGGCTGACAAGAATCGGG
	qKpGA2ox2 Rev	GATGTTCATGTGCAATGGAG
qKdYUC1	*KdYUC1 Forw*	GTCACAATGCCACGCCTTAC
	KdYUC1 Rev	GCAGCCATTTCAGCAGACA
Kd18S	*qKd18S* Forw	AGAAACGGCTACCACATCCAAG
	qKd18S Rev	GACTCATTGAGCCCGGTATTGT

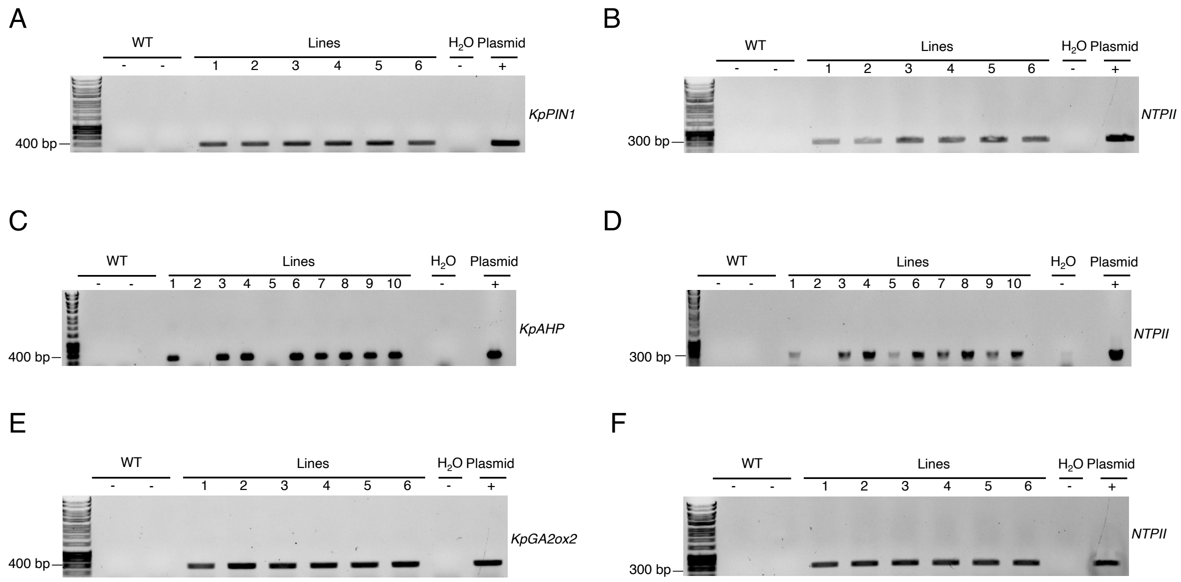

Fig. S1. Genotyping of AS lines. (A), PCR confirmation of the presence of the transgene (A, C, E) and the *NPTII* gene (B, D, F) in independent AS lines of *KpPIN1* (A, B), *KpAHP* (C, D) and *KpGA2ox2* (E, F). Negative control (-): WT and H_2O; Positive control (+): plasmid DNA.

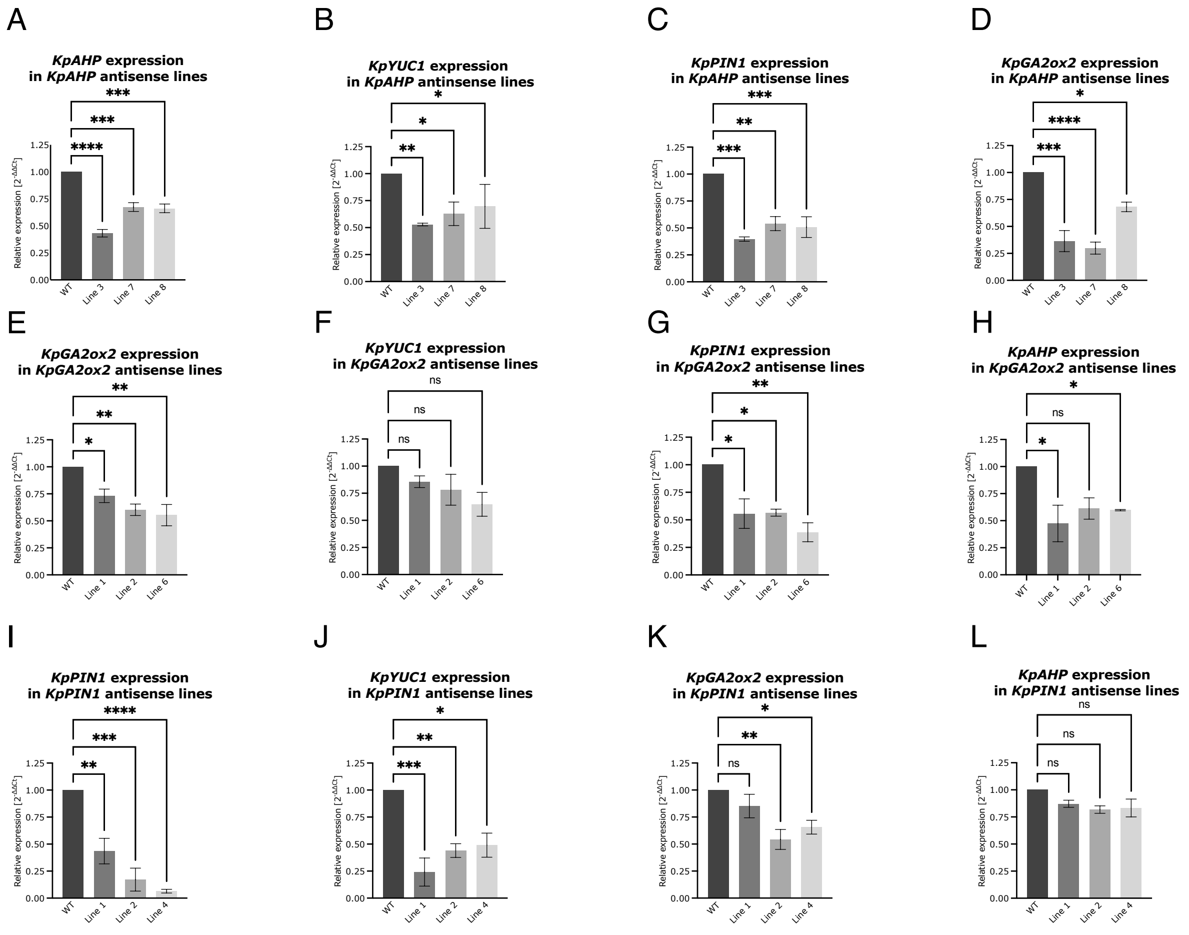

Fig. S2. RT-qPCR analysis of *KpPIN1*, *KpAHP*, and *KpGA2ox2* AS lines. Expression of *KpAHP*, *KpGA2ox2*, *KpPIN1* and *KpYUC1* in *KpAHP* AS lines 3, 7 and 8 (A-D), *KpGA2ox2* AS lines 1, 2 and 6 (E-H), and *KpPIN1* AS lines 1, 2 and 4 (I-L). Comparisons—independent AS lines *versus* WT. One-way ANOVA with Dunnett's Multiple Comparison, ns (non-significant); * (P-value <0.01); ** (P-value <0.001); *** (P-value <0.0002) and **** (P-value <0.0001).
